# Smoking in patients hospitalized for schizophrenia: Prevalence and management challenges

**DOI:** 10.1192/j.eurpsy.2022.2131

**Published:** 2022-09-01

**Authors:** Z. Bencharfa, Y. Amara, S. Belbachir, A. Ouanass

**Affiliations:** University Hospital Center Ibn Sina, Ar-razi Psychiatric Hospital, Salé, Morocco

**Keywords:** schizophrénia, smoking

## Abstract

**Introduction:**

Smoking is the leading cause of preventable death in the world. Studies have shown that the frequency of its use in schizophrenic patients is significantly higher than in the general population,which hinders both treatment strategies and the efficacy of antipsychotics.

**Objectives:**

The objectives of our study are to highlight the prevalence of smoking in this population,to assess their nicotine dependence as well as to support the difficulties of their management.

**Methods:**

We conducted a cross-sectional study of 92 male patients, hospitalized at Ar-razi Hospital in Salé, using the Fagerström scale, associated with a questionnaire that included age, marital status, educational level, somatic comorbidities, current treatment, other substances used, withdrawal attempts, age of first cigarette, family history of smoking, and finally,number of cigarettes per day before and after psychiatric diagnosis.

**Results:**

All our patients were male,the average age was 31 years, 84.8% of our patients were single, 73.9% were without a profession, only 23.9% were under classic neuroleptics while 4.3% were under Clozapine, the most of our patients were also using Cannabis,52.2% attempted a withdrawal, with an average duration of 6 months.69.6% of our patients had a family history of smoking and only 21.7% had somatic comorbidities.The average daily consumption was 12 cigarettes per day before the onset of psychiatric symptoms, rising to 18 cigarettes per day after the psychiatric diagnosis.

**Conclusions:**

The frequency of smoking in schizophrenic patients is high. Unfortunately, these patients remain poorly aware of the harms of smoking, hence the need to integrate a smoking control strategy into the management of schizophrenia.

**Disclosure:**

No significant relationships.

